# Development of the UPSIDES global mental health training programme for peer support workers: Perspectives from stakeholders in low, middle and high-income countries

**DOI:** 10.1371/journal.pone.0298315

**Published:** 2024-02-26

**Authors:** Rebecca Nixdorf, Yasuhiro Kotera, Dave Baillie, Paula Garber Epstein, Cerdic Hall, Ramona Hiltensperger, Palak Korde, Galia Moran, Richard Mpango, Juliet Nakku, Bernd Puschner, Mary Ramesh, Julie Repper, Donat Shamba, Mike Slade, Jasmine Kalha, Candelaria Mahlke

**Affiliations:** 1 Department of Psychiatry and Psychotherapy, University Medical Center Hamburg-Eppendorf, Hamburg, Germany; 2 School of Health Sciences, Institute of Mental Health, University of Nottingham, Nottingham, United Kingdom; 3 East London NHS Foundation Trust, London, United Kingdom; 4 Department of Social Work, Ben Gurion University of the Negev, Be’er Sheva, Israel; 5 The Bob Shapell School of Social Work, Tel Aviv University, Tel Aviv, Israel; 6 Department of Psychiatry II, Ulm University, Ulm, Germany; 7 Centre for Mental Health Law and Policy, Indian Law Society, Pune, India; 8 Butabika National Referral Hospital, Kampala, Uganda; 9 School of Health Sciences, Soroti University, Soroti, Uganda; 10 MRC/UVRI and LSHTM Uganda Research Unit, Entebbe, Uganda; 11 Makerere University, Kampala, Uganda; 12 Ifakara Health Institute, Dar es Salaam, Tanzania; 13 ImROC, Nottinghamshire Healthcare NHS Foundation Trust, Nottingham, United Kingdom; 14 Faculty of Nursing and Health Sciences, Health and Community Participation Division, Nord University, Namsos, Norway; University of Pretoria, SOUTH AFRICA

## Abstract

**Background:**

Peer support in mental health is a low-threshold intervention with increasing evidence for enhancing personal recovery and empowerment of persons living with severe mental health conditions. As peer support spreads globally, there is a growing need for peer support training programmes that work well in different contexts and cultures. This study evaluates the applicability and transferability of implementing a manualised multi-national training programme for mental health peer support workers called UPSIDES from the perspective of different local stakeholders in high-, middle-, and low-income countries.

**Method:**

Data from seven focus groups across six study sites in Africa (Tanzania, Uganda), Asia (India, Israel), and Europe (Germany 2 sites) with 44 participants (3 service users, 7 peer support workers, 25 mental health staff members, 6 clinical directors and 3 local community stakeholders) were thematically analysed.

**Results:**

397 codes were identified, which were thematically analysed. Five implementation enablers were identified: (i) Enhancing applicability through better guidance and clarity of training programme management, (ii) provision of sufficient time for training, (iii) addressing negative attitudes towards peer support workers by additional training of organisations and staff, (iv) inclusion of core components in the training manual such as communication skills, and (v) addressing cultural differences of society, mental health services and discrimination of mental health conditions.

**Discussion:**

Participants in all focus groups discussed the implementation of the training and peer support intervention to a greater extent than the content of the training. This is in line with growing literature of difficulties in the implementation of peer support including difficulties in hiring peer support workers, lack of funding, and lack of role clarity. The results of this qualitative study with stakeholders from different mental health settings worldwide emphasises the need to further investigate the successful implementation of peer support training. All results have been incorporated into the manualisation of the UPSIDES peer support training.

## Introduction

Improving the quality of life of people living with severe mental health conditions is central to personal recovery [1]. A priority of the current global mental health agenda is to address long-term mental health conditions through diverse approaches [2, 3], including person-centred, recovery-oriented services such as peer support [4]. Peer support workers are persons with lived experience of mental health conditions and recovery, who are trained to use this personal knowledge to support other persons with mental health conditions [5]. Peer support aims to provide more user-oriented care, to promote hope, to empower service users, and to destigmatise mental health conditions [6, 7]. Although the overall evidence on the effectiveness of peer support is inconsistent [8], there are promising results demonstrating improvements in recovery-related outcomes including the increase of hope [9], empowerment [8], and self-efficacy [10].

Peer support can be implemented in various settings and services, e.g. in outpatient and inpatient services [11], assertive community programmes [12], case management [13], and crisis resolution teams [14], Showing promising results in the recovery and empowerment of persons with severe mental health conditions in these various contexts, peer support is now an established intervention in the policy of different high-income countries [15] and increasingly implemented in a variety of different settings in low- and middle-income countries (LMIC) [16, 17]. In their meta-analysis of 14 studies, Vandewalle and colleagues [18] analysed effects of peer support in LMIC on depression-related and PTSD-related outcomes and found small to medium effect sizes. Peer support can be a helpful approach to closing the mental health care gap [19] and is increasingly implemented worldwide [17]. Nevertheless, peer support in LMICs is under-researched [20].

To improve the chances of a successful implementation of interventions in study sites with different cultural and socio-economic settings, modifications to these interventions need to address local contextual and cultural aspects [21]. Therefore, it is important to ensure applicability and transferability of an intervention [22]. Applicability refers to whether an intervention can be implemented in the local setting, while transferability refers to whether the implementation of an intervention would show similar effectiveness detected in the original study setting [23]. Established implementation frameworks such as the Consolidated Framework for Implementation Research and the Culturally Responsive Evaluation also consider modification of the intervention to fit specific contexts [24]. A systematic review [25] on modifications of peer support interventions identified that modifications can be made in relation to role expectations, initial training, type of contact, role extension, workplace support for peer support workers, and recruitment. Nevertheless, best practice examples from the field of peer support on modifications to ensure successful local implementation are missing [17].

Training and supervision of peer support workers increase fidelity of peer support interventions [26]. An online survey of a national sample of 597 peer support workers in the US showed that more than half of the peer support workers received between 20 and 80 hours of training including a variety of different training contents [27]. In this study the perception of having had sufficient training and the length of training predicted job satisfaction. A qualitative process evaluation of a complex intervention to enhance personal recovery demonstrated a need for effective training [28]. Although peer support training is important in the successful implementation of peer support in mental health care systems [29], and peer support in mental health care is implemented globally, there is a lack of culturally sensitive training to prepare peer support workers in different settings.

A global research consortium named Using Peer Support In Developing Empowering Mental Health Services (UPSIDES) was launched in 2018 to address the difficulties described in the implementation and evaluation of peer support in global mental health systems. UPSIDES is a 5-year multicenter study conducted in Africa, Asia, and Europe, with the aim to implement, evaluate, and scale up a peer support intervention for persons with severe mental health conditions in low-, middle- and high-income settings (www.upsides.org) [30]. As part of the study, a training programme for peer support workers was developed and adapted regarding its applicability and transferability for different cultural settings.

This study aims to explore stakeholders’ perceptions of the UPSIDES peer support training concept and applicability in various settings.

## Materials and methods

### Design

The evaluation of the preliminary concept for global mental health peer support worker training was realised through a participatory, qualitative design using thematic analysis. Seven focus group discussions were conducted to understand common and individual requirements for a globally applicable peer support programme. The report of the study is guided by the 32 items of the consolidated criteria for reporting qualitative studies [COREQ, 31]. All participants provided written informed consent. The study was approved by the ethics committee in each of the respective countries including Ulm University Ethics Commission (Application nr. 195/18), Ärztekammer Hamburg, Germany (MC-230/18), Mengo IRB Uganda (MH: 360; MH/REC/141/8/2018), National Institute for Medical Research Tanzania (NIMR/HQ/R.8a/Vol.IX/2982), Institutional Review Board, Ifakara Health Institute, Tanzania (IHI/IRB/No. 28–2018), Human Subjects Research Committee of Ben-Gurion University (ref: 1621–2), Indian Council of Medical Research (Indo-foreign/66/M/2017-NCD-1) and Indian Law Society (ILS/37/2018). Five research questions were established:

RQ1 How applicable is the training to sites in different contexts of the world?

RQ2 What adjustments need to be made to the training to adapt to different contexts?

RQ3 What are the common characteristics (strengths and weaknesses) of the training across the sites?

RQ4 What is missing in the training?

RQ5 Which cultural differences are relevant to implementation of training?

### Preliminary global mental health peer support training concept

The preliminary training concept and a preliminary training manual were developed by RN, CM and JR based on (a) systematic reviews regarding best practice for implementation, modification and training in peer support [25, 29], (b) a situational analysis on previous experiences with peer support at the different study sites [29], (c) a conceptual framework of key principles of peer support developed by the UPSIDES consortium [32] derived from a researcher-led integration of established core principles from high-resource settings, (d) peer support training manuals previously implemented by members of the UPSIDES consortium, including the Brain Gain Project (Butabika, Uganda [33]), the ImROC training (UK [34]), the EX-IN curriculum (Germany [35]), the WHO’s QualityRights programme implemented in Gujarat, India (QualityRights Gujarat, India [36]), the Healthy Options programme (Tanzania [37]), the P2P training (UK [38]) and the Yozma Derech-Halev, Consumer-provider training programme (Israel [39]), The training aimed to provide peer support workers with a shared understanding of peer support and recovery, and offer reflection about the future role as a peer support worker [40]. The initial training concept included five core training modules: Recovery, Peer Support, Planning & Community, Communication Skills, and Peer Support Worker Role & Network. During the training, a variety of different learning methods were used, e.g., presentations, discussions, role plays, and exercises. For each topic, examples of good practice were used to prompt a discussion, and all training participants were encouraged to draw from their own experiences and ideas, to create a shared knowledge, based on the whole group’s experiences. The training was planned to last five days with eight hours of training per day, whereby the time schedule was flexibly adapted to local conditions.

### Participants

The research staff of each study site in Beer Sheva (Israel), Dar es Salaam (Tanzania), Ahmedabad (India), Hamburg (Germany), Kampala (Uganda) and Ulm (Germany) identified key local stakeholders playing a central role in the implementation and evaluation of the peer support intervention and purposively invited them to the local focus group. The recruitment period lasted from 12/2018–01/2019. Potential participants were informed about the UPSIDES project and the objectives of the focus group in a personal conversation, by phone, email, or post. Inclusion criteria were having at least three months of experience of (i) employing peer support workers, (ii) working as a peer support worker, (iii) working as a peer trainer, or (iv) with mental health conditions in the self-report of service users. The research team judged three months is a threshold because participants should have had a minimum of experience with peer support. For study sites where peer support had not been implemented yet, participants to be included needed to be planning or having interest in (i)-(iii). Exclusion criteria were being under 18 years of age, and being unable to give informed consent.

### Procedure

Each focus group was facilitated by one or two UPSIDES researchers from Israel, Tanzania, India, Uganda or Germany with a degree in psychology, social work, or allied fields, and a student assistant who took field notes during the discussion, where possible. Only the facilitators and participants were present. The focus groups were held in the local languages and took place in different locations, including clinical, outpatient, or university settings. A semi-structured focus group guide was developed by the UPSIDES research team in Hamburg (RN and CM). The focus group guide (S1 File) included an instruction to welcome the participants, explain the main ethical study formalities, open the discussion, and demonstrate exemplary questions. First, the researchers presented the UPSIDES training concept. The presentation included a detailed overview of the contents of the individual training modules and the corresponding exercises. Participants were then given an opportunity to ask questions, which were immediately clarified by the facilitators to ensure that all participants have similar understanding. The training concept was discussed using the following sample questions:

‘Could the training be held in this context?’ (RQ 1)
‘What changes need to be made?’ (RQ 2)
‘What do you think about the training for peer support workers?’ (RQ 3)
‘Are any core elements missing?’ (RQ 4)
‘What needs to be done, to adapt the training in this local context?’ (RQ 5)

In total, the focus groups were scheduled for 90 minutes. All focus groups were audio recorded, transcribed, and translated to English by research workers bilingual in the local language and English. The translation team of the UPSIDES consortium checked all translated transcripts for comprehensibility and content equivalence [41]. The seven focus groups reached a point of saturation: the study team agreed that the data was sufficient to answer the research questions, and more focus groups would not yield any additional information to answer the research questions [42].

### Analysis

The transcripts were analysed with MAXQDA using thematic analysis [43]. The analysis was conducted by RN (research assistance in Hamburg, Germany) together with a student assistant (Hamburg, Germany) with consultation by YK (UK). In terms of positionality and reflexivity, both coders were young-aged white woman, educated in the field of psychology and socialised in a high-income country [44, 45]. Both coders were employed at a German University clinic. RN is a PhD candidate, whose research focuses on recovery, peer support, and prevention of coercion. YK is a senior male researcher, oriented to Asia. Throughout the development of focus group guidelines, data collection and data analysis colleagues from LMICs were consulted.

First, the researchers repeatedly read the transcripts to familiarise themselves with the data. Second, initial codes were generated. To guarantee internal consistency, all transcripts were coded by two researchers and codes were discussed to generate consensus. The coding was theory-driven and led by the research questions [46]. Overall, 397 codes (S2 File) were identified (e.g. ‘communication skills’, ‘hospital policies’, ‘ability to disclose’). In a third step, the codes were visualised using a mind map (S3 File) to categorise them into themes [43]. In a fourth step, the themes were reviewed and refined by RN and YK to establish thematic coherence and to generate distinctive themes. In a last step, the themes were defined, renamed and subthemes were generated. The research team reviewed and agreed with the finalised themes and subthemes.

The thematic analysis and mind mapping of the codes lead to seven initial themes. These themes were reviewed and refined to establish thematic coherence and to generate distinctive themes, guided by the research questions. The refinement resulted in five main themes: (i) enhancing applicability through better guidance and clarity of training programme management, (ii) adjustments should include more detailed and interactive learning content, (iii) additional training to enable organisational system change, (iv) key training modules include communication, lived experience, rights, self-care and skills, and (iv) mental health systems priorities, societal norms, and social inequities influence the training.

## Results

### Participants

A total of 44 participants took part in seven focus groups, comprising 3 service user representatives, 7 peer support workers, 25 mental health staff members, 6 clinical directors and 3 local stakeholders (relative, religious representative, politician). The focus groups were held at each of the six UPSIDES recruiting study sites in Beer Sheva (Israel), Dar es Salaam (Tanzania), Ahmedabad (India), Hamburg (Germany), Kampala (Uganda) and Ulm (Germany). The sociodemographic characteristics of the focus group participants are displayed in Table 1.

**Table 1:** Sociodemographic characteristics of focus group participants.

		Study site						Total
		Beer Sheva (Israel)	Dar es Salaam (Tanzania)	Ahmedabad (India)	Hamburg (Germany)	Kampala (Uganda)	Ulm (Germany)	
		n = 8	n = 8	n = 5	n = 8	n = 5	n = 10	n = 44
Age		42	31.3 (7.3)	42.4 (8.8)	31.5 (9.2)	42.4 (7.5)	53.4 (6.9)	40.5 (8.3)
	M (SD)							
Gender								
	female	n = 6	n = 4	n = 2	n = 5	n = 3	n = 6	n = 26
	male	n = 2	n = 4	n = 3	n = 3	n = 2	n = 4	n = 18
	diverse	n = 0	n = 0	n = 0	n = 0	n = 0	n = 0	n = 0
Role								
	service user	n = 1	n = 0	n = 0	n = 2	n = 0	n = 0	n = 3
	peer support worker	n = 1	n = 0	n = 0	n = 4	n = 1	n = 1	n = 7
	mental health worker	n = 2	n = 8	n = 5	n = 1	n = 3	n = 6	n = 25
	local stakeholder	n = 0	n = 0	n = 0	n = 0	n = 0	n = 3	n = 3
Experience with peer support								
	yes	n = 8	n = 2	n = 5	n = 8	n = 3	n = 8	n = 34
	no	n = 0	n = 6	n = 0	n = 0	n = 0	n = 2	n = 10

### Themes

The five main themes were identified to answer the research questions on applicability (RQ1), adaptation (RQ2), strength and weaknesses (RQ3), missing contents in the training concept (RQ4), and cultural differences (RQ5), as shown in Table 2. Subthemes were added to reflect the individual aspects of each theme. The data extracts that comprised Theme 1 ‘Enhancing applicability through better guidance and clarity of training programme management’ showed that although the training was rated as helpful overall, more guidance on the implementation of training and sufficient resources for the facilitation of the training was necessary to enhance applicability of the training (corresponding to RQ1). Data extracts from Theme 2 ‘Adjustments should include more detailed and interactive learning content’ showed that there should be enough time for training to allow participants to reflect the lived experience and to practice the peer support worker role with local examples in interactive role plays (RQ2). Data extracts from Theme 3 ‘Additional training to enable organisational system change’ showed that negative attitudes towards peer support workers need to be addressed by additional training of organisations and staff (RQ3). Data extracts from Theme 4 ‘Key training content includes communication, lived experience, rights, self-care and skills’ highlighted necessary topics for a core training for peer support workers (RQ4). Data extracts from Theme 5 ‘Mental health systems priorities, societal norms, and social inequities influence the training’ showed that cultural differences of society, mental health services and discrimination of mental health conditions have an influence on the training implementation (RQ5).

**Table 2:** Research questions, themes, and illustrative quotes.

	Research Question	Theme	Subthemes	Illustrative Quote
1	Applicability	Enhancing applicability through better guidance and clarity of training programme management	1.1 Benefits of the training 1.2 Selection criteria for participation 1.3 Employment conditions 1.4 Resources for implementation	*“I think the selection for such a training is quite important*. *You should do some good thinking about how to select people*.*”* [Ulm, Germany]
2	Adaptation	Adjustments should include more detailed and interactive learning content	2.1 Duration of training 2.2 Interactive learning methods 2.3 Accessibility	*“In that time frame*, *can we have a room for an extension*? *In any case*, *we just plan ahead that we might finish in time or not so in our planning we should create time for that extra time in case it’s needed*. *So we just plan and think ahead that we might need more time”* [Uganda]
3	Common characteristics (strengths and weaknesses)	Additional training to enable organisational system change	3.1 Organisational change 3.2 Attitudes about peer support worker 3.3 Training for organisations and staff.	*“One does a training or apprenticeship and then they are left alone and then things like that happen*, *that they get frustrated*, *or clinics don’t want to change their framework and the peer support workers also run up against the wall with their ideas and then in the worst case*, *that they are laid off again*.*”* [Ulm, Germany]
4	Missing contents in the training concept	Key training content includes communication, lived experience, rights, self-care and skills	4.1 Communication 4.2 Lived experience 4.3 Rights and advocacy 4.4 Self-care 4.5 Skills	*“Models that need to be added that encourage the individuals that I accompany to reduce their use of medication- training*, *knowledge*, *a body that has knowledge provided by the peer professionals*. *That is missing for me*.*”* [Israel]
5	Cultural differences	Mental health systems priorities, societal norms, and social inequities influence the training	5.1 Perspectives on mental health 5.2 Community involvement 5.3 Social inequities	*“[…] but then I also think that the stigma of mentally ill people is perhaps an international issue after all*.*”* [Hamburg, Germany]

### Theme 1: ‘Enhancing applicability through better guidance and clarity of training programme management’ (RQ1)

Theme 1 covers all aspects that participants considered necessary to improve the applicability of the training at the different study sites. In Theme 1, four subthemes were identified, including ‘benefits of the training’, ‘selection criteria for participation’, ‘employment conditions’, and ‘resources for implementation’.

#### Benefits of the training

Participants suggested the training would be helpful for their contexts as it was seen as a preventive, low-threshold intervention. Peer support worker were described as potential role-models who can empower service users and who can enable a change of perspectives within the mental health systems.

*“According to what I see within the training manual*, *it looks very empowering to Peer Support Workers*. *It helps them to identify their roles as Peer Support Workers and help them to understand what recovery is to them and it will help them also like to use their own stories to empower others or support others*, *so for me that’s what I realise it’s going to be*.*”* [Uganda]

#### Selection criteria for participation

Despite the anticipated benefits, participants in all focus groups gave clear suggestions that applicability of the training would be enhanced by more clarity of training implementation. Participants expressed uncertainty who should participate in the training and what access requirements should be set. Participants suggested mental health stability, level of recovery, level of education, and personal motivation, as selection criteria. In some focus groups participants raised concerns if participants with lived experience of mental health conditions are able to participate in a training.

*“The more educated they [training participants] are*, *the better but even minimum education is okay*.*”* [India]

“*You have to be really strong for yourself and I think that someone who decides to become a [peer support worker] feels the strength but if you’re speaking about everyone*, *there needs to be some sort of recovery measure*. [Israel]

*“So*, *even there I would still make a selection*. *So that I see that we have motivated people*.*”* [Ulm, Germany]

#### Employment conditions

Participants from three sites (Israel, Hamburg and Ulm (Germany)) expressed a need for a clear job and employment perspective for training participants and clarity of the scope of the peer work. The trainings should offer the participants a certificate. Participants wished for clarity regarding the salary for peer support workers after the training programmes.

*“And the question is*, *who pays them*, *how does they pay*?*”* [Hamburg, Germany]

*“Most of the times*, *there is no budget*.*”* [Ulm, Germany]

#### Resources for implementation

Three sites (Uganda, Tanzania, and Ulm (Germany)) reported that they were lacking resources for training delivery, including the environment to set up the training (rooms and materials), compensation for transport costs for participants, enough time and space for breaks, and resources like food and drinks.

*“I was thinking about the same thing that we will be having different sessions and different groups*, *the only challenge is on the place to conduct those sessions […]*.*”* [Tanzania]

*“Then that would mean that there should be some facilitation to bring them because they can’t stay throughout a day without something*.*”* [Uganda]

Overall, Theme 1 shows that applicability for training in different settings is increased when clarity of training implementation including selection criteria for participation and sufficient resources for implementation are available.

### Theme 2 ‘Adjustments should include more detailed and interactive learning content’ (RQ2)

Theme 2 includes important adjustments that have been proposed to improve the training. In Theme 2, three subthemes were identified, including ‘duration of training’, ‘interactive learning methods’, and ‘accessibility’.

#### Duration of training

Participants from all focus groups stated that the training seemed too short to them, and they recommended to extend the training and to include frequent breaks, regenerative elements, and refresher sessions to let the learning experiences sink in.

*“Well*, *it’s also clear to me that it’s much too short*.*”* [Hamburg, Germany]

*“It should continue for more time*, *and it should be maintained because we are doing this step by step*.*”* [India]

*“I would say it should be the length of a semester in order that it be done properly*. *I think that it takes a certain amount of time until things sink in*, *and a person feels comfortable with what he is learning*.*”* [Israel]

*“I would find it more appropriate to do half a day a week and then draw it out accordingly and leave some modules for after the activity has started*.*”* [Ulm, Germany]

#### Interactive learning methods

Participants from four sites (India, Israel, Ulm (Germany), and Uganda) recommended to include more interactive role plays or simulations and to practice the peer support worker role with local examples. Participants from Hamburg (Germany) recommended to include more reflection sessions.

*“And I think we’re already back to the question of whether it’s actually possible to depict this in five days*, *for example*, *maturing*, *co-developing*, *encounters*, *reflection*, *whether there’s actually any room for it*?*”* [Hamburg, Germany]

*“Role play might make it easier to understand*.*”* [India]

#### Accessibility

Participants from four sites (Hamburg (Germany), India, Tanzania, and Uganda) recommended to simplify the training content to enable practical learning.

*“That might be very general*. *We should keep it specific to whatever is related to patients*.*”* [India]

*“It should be simple and clear it should not be complicated*.*”* [Tanzania]

In conclusion, Theme 2 shows that peer support training needs sufficient time, should include interactive learning methods, and should be designed to be easily accessible through simple and practical exercises.

### Theme 3 ‘Additional training to enable organisational system change’ (RQ3)

Theme 3 focuses on the strengths and weaknesses of the training and what recommendations the participants derive from it. In Theme 3, three subthemes were identified, including ‘organisational change’, ‘attitudes about peer support worker’, ‘training for organisations and staff’.

#### Organisational change

Participants in all focus groups pointed out the potential of training peer support workers to enable a change in the mental health systems towards more service-user orientation and recovery focus.

*“People don’t understand psychiatric symptoms*. *They don’t understand*, *if they haven’t experienced it*. *The second you experience it you can understand me*. *You give a good word*, *when I’m in mania how do I get out of mania*, *how to help yourself*. *Psychiatrists aren’t there*, *the know from the book*, *but this information isn’t completely accurate*, *unfortunately*. *One big philosophical question*.*”* [Israel]

#### Attitudes about peer support worker

At the same time, in all focus groups negative attitudes and reservations about peer support workers were raised, including negative experiences with staff and peer support workers.

*“I sometimes try talking with professionals about how their job is different from ours*, *from people with knowledge from experience*, *and I try to explain that these two jobs do not negate one another—I felt hostility*.*”* [Israel]

*“One often meets resistance from both sides*. *From one side*, *from the professionals*, *they say*, *are you going to stick your nose into my job*? *But resistance can also come from patients*, *so*: *“Why are you now presuming to tell me this*? *You’re no better than me or different or you’re just as sick*.*””* [Ulm, Germany]

#### Training for organisations and staff

Participants from three focus groups (India, Israel, and Ulm (Germany)) recommended an additional training for organisations and staff to overcome negative attitudes against peer support workers. In addition, support for the peer support workers start in the work with work placement procedures, internship, regular supervision, and collegial counselling was recommended.

*“I am very excited about this seminar*. *[…] There needs to be training for the places that are hiring peers*. *Every position of a peer specialist [peer support worker] is within a workplace and so the training is crucial in order that this be successful*.*”* [Israel]

Overall, Theme 3 shows that changes in organisations through peer support are only possible if the organisations also undergo training on recovery and peer support. In addition, peer support workers need to be supported on the job, as negative attitudes from other staff are likely.

### Theme 4 ‘Key training content includes communication, lived experience, rights, self-care and skills’ (RQ4)

Theme 4 covers relevant content for the core training for peer support workers and which topics should have special focus in the training. Participants from all focus groups stated that important topics are missing or need to be addressed in more detail in the core training. In Theme 4, five subthemes were identified, including ‘communication’, ‘lived experience’, ‘rights and advocacy’, ‘self-care’, and ‘skills’.

#### Communication

In three focus groups (Tanzania, Ulm (Germany), and Uganda) participants suggested to focus more on communication and to include more exercises on active listening and non-violent communication.

*“The communication skills has been scheduled for the fourth day instead of the first day because it is something which you are supposed to learn before moving forward and it is good because most people face difficulties in talking*.*”* [Tanzania]

*“some kind of tools are [needed]*, *such as non-violent conversation*, *different tools for language that can be used”* [Ulm, Germany]

*“Because if you bring active listening*, *communication some where there*, *so what are you going to communicate when we are talking about peer support*? *Is it something we need first*? *People to understand communication*, *so that as they are discussing these ones we are already at par*, *even when they are discussing peer support and planning*, *they have learnt about communication because all this is about communication*.*”* [Uganda]

#### Lived experience

Participants from three focus groups (Israel, Hamburg and Ulm (Germany)) recommended to focus more on the lived experience and to teach training participants how to reveal their lived experience and when and how to share their own recovery story.

*“What am I and when do I self-disclose*? *The story has to be well-processed in order for him to be able to use it in front of others*. *And he also needs to know that there are aspects that he shouldn’t touch upon and shouldn’t use*. *There are topics that are still very sensitive*.*”* [Israel]

*“It’s also biographical work and stories like that*.*”* [Hamburg, Germany]

*“What is incredibly important in the present training that we have here is the confrontation with one’s own recovery history*. *I don’t think they all succeed equally well*, *yes*. *So*, *I think good PSWs are characterized by the fact that they are aware of their recovery history*, *what was helpful there*, *what happened there*, *that they are also aware of what they have to look out for in themselves*. *And I think*, *to be honest*, *that’s not something you can learn in two days*.*”* [Ulm, Germany]

#### Rights and advocacy

Participants from three focus groups (Israel, Hamburg and Ulm (Germany)) recommended to include a training module on rights and advocacy to enable peer support workers to advice and empower service users. Participants from the focus groups in Israel and Ulm (Germany) suggested to focus specifically on the reduction of medication. This suggestion contrasts a statement from a participant from Uganda, who stated that peer support workers should help service users to stick to their medication.

*“So*, *if there was justice in it*, *for example*?*”* [Hamburg, Germany]

*“People need to know that they have the right to determine and decide what they take and why not*, *except for in extreme cases*.*”* [Israel]

*“I consider this to be a very important element of peer support*, *also with a view to the patients*, *where a lot*, *I find*, *takes place in the consultation*, *so*, *based on the motto*, *stand up for your rights*, *discuss with the doctor again about your medication*, *if you do not agree with it*, *yes*.*”* [Ulm, Germany]

#### Self-care

Participants from three focus groups (India, Tanzania, and Ulm (Germany)) recommended to include self-care and ground rules into the core training, and to develop clear job descriptions and guidelines for the peer support work, enabling training participants to protect themselves from burnout during their work as peer support workers.

*“They have a job description*, *but you know where the variation comes*? *I will tell you where the variation comes*. *In my experience*, *they get allotted other work in the wards*. *Then I have to go and tell them that this is not their work*.*”* [India]

*“There should be ground rules so that people can know what to do and not what to do*.*”* [Tanzania]

*“I think it’s a good example to tell [peer support workers] that they have to take care of themselves*. *That you have to check*: *How I’m doing right now and what I believe I’m capable of or not*.*’*.*”* [Ulm, Germany]

#### Skills to enhance core training

Participants from five focus groups (India, Tanzania, Uganda, Ulm and Hamburg (Germany)) recommended to include specific skills into the core training. Participants from India, Tanzania, Uganda, and Ulm (Germany) recommended to expand the module to include goal setting, problem solving and conflict management.

*“We should focus more on the second day training about setting goals*. *It is more important*.*”* [India]

*“I think that there is one component of problem-solving*, *it is a very good component because when people meet many things are discussed*, *they discuss about the problems which they are facing so the problem-solving approach will show how to solve those problems*.*”* [Tanzania]

*“I was just thinking that the approach of problem solving*, *then I was also thinking about having some kind*.., *the component of conflict resolution because this one will help them also to resolve their own conflicts and also somehow to guide the peers elsewhere*, *their families*, *caretakers when they get there when they are offering support*, *it will guide them somehow*.*”* [Uganda]

Participants from Hamburg (Germany) recommended to include a module on trauma-focused peer support.

*“And trauma*. *But church*, *anti-stigma would then be appropriate again*. *But that would be another new step that you have to include*, *also in research*.*”* [Hamburg, Germany]

Overall, Theme 4 recommended the following topics for the core training: communication, lived experience, rights and advocacy, self-care, as well as specific skills for conflict resolution, goal setting and trauma-specific peer support.

### Theme 5 ‘Mental health systems priorities, societal norms, and social inequities influence the training’ (RQ5)

Theme 5 covers relevant cultural perceptions that may have an additional influence on the delivery and implementation of the training in different settings. In Theme 5, three subthemes were identified, including ‘perspectives on mental health’, ‘community involvement’, and ‘social inequities’.

#### Perspectives on mental health

Within and between all focus groups different perspectives on mental health and mental health conditions were offered. Participants expressed medical perspectives on mental health conditions labelling service users (and peer support workers) as ‘patients’ who are ‘ill’. Some participants recommended medication as the preferred treatment. Other participants offered a recovery-focused perspective on mental health with an understanding of mental health conditions as ‘a normal reaction to extreme circumstances’.

*“Perhaps it is related to trauma—a situation that is not normal that led to a normal reaction*, *that led to a circumstance that wasn’t normal*. *A victim of misunderstanding*. *Not necessarily a mental illness*.*”* [Israel]

*“The way we are working with the patients there are two or three cultural dynamics*, *there are those who come and the patient is in a confined space*, *area or context*. *There are those who are in recovery stage but are in the middle-class contexts and there are those who are in the upper class so when you were presenting*, *I was trying to go back and think about my patients*, *and they are in those stages*.*”* [Tanzania]

*“What do we do when someone says it’s a metabolic disorder*? *Medication helps*. *I’ve had clients like this*. *The little pink pill helped*, *and I don’t care about anything else*. *You still have to learn to live life again*.*”* [Ulm, Germany]

*“There are some people who keep that sick role*.*”* [Uganda]

#### Community involvement

Participants from three focus groups (India, Tanzania, and Uganda) expressed a high relevance of the community in the society and recommended to include relatives and family members of training participants during training implementation.

*“For the community*, *priority is the patient*. *They are their guardians*, *parents or whatever*. *So*, *it is good if they are involved to encourage the patients*.*”* [India]

*“They [family members] are the ones to allow them [training participants] and support them financially and socially and so they should have some information to get to know and to be aware that there is maybe a training*.*”* [Uganda]

#### Social inequities

Participants from all focus groups stated that people with mental health conditions are facing social inequities including (self-)stigma, discrimination, isolation, loneliness, and that the peer support should focus on social inclusion.

*“But whenever we ask patients about their goals*, *we usually only get two answers*: *marriage and employment*.*”* [India]

*“There is the issue of stigma which persists on the patient and the community so I was thinking have you thought of a way to acquire them because there is a patient who is in the lower economic class and could have been a good peer supporter*, *have you thought of how you can join them to be peers*?*”* [Tanzania]

*“Well*, *I think people with severe mental illness who are residual for example*, *are often very lonely*. *They often suffer from loneliness and they say*, *I cannot go anywhere alone*, *if I only had someone to go with me*. *Cycling for example*. *We can go cycling together*, *or swimming*.*”* [Ulm, Germany]

Overall, theme 5 shows different attitudes towards mental health which is why different understandings of mental health within the different services can be assumed. A second influence is the extent of community involvement of the society of the respective setting. The discrimination and isolation of people living with mental health conditions was named as a common social challenge, hence a focus on social inclusion seems appropriate in all settings.

## Discussion

This qualitative study evaluated a training concept for mental health peer support in high-, middle-, and low-income settings, to further improve the UPSIDES peer support training manual. Five implementation enablers were identified: (i) Enhancing applicability through better guidance and clarity of training programme management, (ii) provision of sufficient time for training, (iii) addressing negative attitudes towards peer support workers by additional training of organisations and staff, (iv) inclusion of core components in the training manual such as communication skills, and (v) addressing cultural differences of society, mental health services and discrimination of mental health conditions.

Interestingly, participants in all focus groups discussed the implementation of the training and peer support intervention to a greater extent than the content of the training. This is in line with growing literature of difficulties in the implementation of peer support including difficulties in hiring peer support workers, lack of funding, and lack of role clarity [47–49]. Participants in all focus groups expressed uncertainty about the criteria for selection of training participants. Uncertainty about the role of peer support workers was evident here, which is repeatedly discussed in peer support research [29]. A longer training duration was seen as necessary in all focus groups to ensure adequate preparation for the work as a peer support worker. Overall, the length of different peer trainings varies greatly [27], although studies often do not report sufficient details about peer trainings [9, 27] which makes it difficult to compare them. Peer support workers repeatedly report they are confronted with stigma from other staff at work [50, 51]. These attitudes were reported across sites in all focus groups or were evident from the participants’ choice of words. This highlights the need to prepare organisations and staff for peer support in their own training and to continuously support peer support workers through supervision [29]. Possible lack of resources was mentioned as a possible barrier to implementation in both low-income settings as well as in one high-income setting, which highlights the relevance of sufficient funding [48].

Recommendations regarding the content differed considerably between the focus groups. This highlights the different contexts of the study sites and the need to adapt the training to these contexts [52]. This became particularly clear with the topic rights and advocacy. In the Israel focus group, it was recommended to add a module on medication withdrawal, while participants from Uganda wanted the peer support workers to help the service users to take their medication regularly. A participant from Ulm (Germany) also expressed concern that the peer support workers might contradict the hospital’s internal focus on medication. To respond to the differences in content, we added optional, site-specific additional modules to the UPSIDES core training. Based on the presented data, the topics of recovery, lived experience, communication, and self-care were seen as necessary for the core training, which is in line with an international expert consensus [53].

A difference between the low- and middle-income settings and the high-income settings was community involvement. This topic was mentioned exclusively in the Indian, Ugandan, and Tanzanian focus groups. For implementation in collectivist vs. individualist societies, the inclusion of important reference persons such as relatives and family members in training planning and implementation should be considered. In UPSIDES, the inclusion of a wide range of stakeholders at all study sites was vital to improving the UPSIDES training. Before delivery of the training in a train-the-trainer workshop in Tanzania in 2019, from which trainers went to their study sites to train future UPSIDES peer support workers, results of this study were incorporated in the training manual, by including clear guidance of the implementation process, stressing the need for sufficient resources and preparation of the organisations who will be employing UPSIDES peer support workers, and adding further training modules with a special focus on site-specific requirements.

The results of the focus groups were incorporated into the preliminary training design, which resulted in the manualized field version of the training [40], that is available on the website (www.upsides.org). The resulting training and the associated peer support intervention were piloted [54] and tested in a randomised controlled trial [55].

### Strengths and limitations

This study assessed the perspective of different stakeholders from high-, middle-, and low-income settings. Using a qualitative approach allowed for a deeper understanding of local factors influencing the implementation of a peer support training. Nevertheless, sample size was small and the perspective of service users and peer support workers was under-represented. All coders and analysts were researchers from high-income settings, which might not have appropriately collected and interpreted the data [56]. To address this, the data material was double-coded, and the codes were discussed with a researcher from another study site (UK). However, future research should employ more culturally aware strategies for data collection and interpretation (e.g., communication style differences [57]).

## Conclusion

By involving important stakeholders in the development of the global UPSIDES peer support training, five implementation enablers were identified for the implementation of peer support training in different settings in mental health systems: (i) Enhancing applicability through better guidance and clarity of training programme management, (ii) provision of sufficient time for training, (iii) addressing negative attitudes towards peer support workers by additional training of organisations and staff, (iv) inclusion of core components in the training manual such as communication skills, and (v) addressing cultural differences of society, mental health services and discrimination of mental health conditions. The field version of the UPSIDES training manual aptly reflected the expertise of people with a lived experience of mental health conditions, as well of important partners who are vital to the successful execution of peer support. The results are particularly important as peer support is implemented in a variety of different settings, thus enhancing the need for culturally adaptable peer training programmes [17].

## Supporting information

S1 File Focus group guide. (PDF)

S2 File Complete list of codes. (PDF)

S3 File Mind map. (PDF)
