# Supplementary material for: Development of the UPSIDES global mental health training programme for peer support workers: Perspectives from stakeholders in low, middle and high-income countries
Source: PLoS One. 2024 Feb 26;19(2):e0298315. doi: 10.1371/journal.pone.0298315 (PMC10896522; doi:10.1371/journal.pone.0298315)
Supplement: S1 File — (PDF) [file pone.0298315.s001.pdf]

WP3 Focus Groups<br>Preliminary adaption preparation<br><br>Topic-Guides

WP3	Task lead: Candelaria Mahlke & Julie Repper	Preliminary adaption preparation
2.1	Focus Groups	12/18 – 01/19

Aim: The aim is to identify mismatches between the generic/core intervention and corresponding training components, materials and our partners' experience of peer support intervention and training and the features of intended applications within the countries.

The Focus Groups are conducted

- to discuss the preliminary training manual
- to discuss if the planned training is culturally appropriate and meet the site-specific requirements
- to discuss if there are any aspects missing in the material

Modalities:

One focus group will be conducted at each recruiting site in **December** 2018 or **January** 2019. The focus group will comprise 4 to 11 participants and will consist of an approximately **30-minute presentation** and **60-minute discussion**. The Focus group will be recorded, immediately transcribed and anonymized by removing any identifying information about participants or named third parties, translated if needed into English.

Participants:

Inclusion Criteria:

4 – 11 local stakeholders with at least 3-month experience of

(1) employing peer support workers (where available) (2) working as peer support workers (where available) (3) peer trainers (where available) (4) service user

If not available, local stakeholders with planning or having interest in

(1) employing peer support workers (2) working as peer support workers (3) working as peer trainer (4) potential service user

Overview: Procedure

(1) Welcome the participants (**ca. 5 Min.**)
(2) Fill out the informed consent (No. 2.1) and the participant data sheet (**ca. 5 Min.**)
(3) Presentation of the training manual (**ca. 30 Min.**)
  → Start of the focus group: Start the audio recorder
(4) Clarify questions about the materials (**ca. 15 Min.**)
(5) Discussion of the materials (**ca. 35 Min.**)

Instructions

Part 1: Introduction and presentation

Welcome everybody to our focus group. My name is ____ and this is my colleague _____.

Thank you for taking the time to discuss with us today our preliminary Peer Support training for the UPSIDES project.

The focus group will take about 90 minutes.

At first, we would like to present you the training manual. We present you the core training elements. This should be locally adapted at each site.

The presentation will take about 30 minutes and if you want to you are welcome to take notes.

Afterwards, we will clarify any questions that might come up during the presentation.

Also, we would like to hear your opinion and thoughts on the training. We would like to hear from your experience, if you think the planned training for Peer Support Workers would be feasible in this context.

The discussion will take about 60 minutes.

All participants need to provide Informed consent in order to participate in the focus group. Everything you say during the focus group will be anonymized and we ask everybody to please keep everything said confident.

Before we begin, I would like to know if there are any questions so far?

→ Fill out the **informed consent** and the **Participant Data Sheet**

→ Present the training manual with support of the Power Point Presentation. (See presentation of the training manual)

Instructions

Part 2: Focus Group

After you presented the training manual:

Thank you for your attention.

Now we can start with the actual focus group discussion.

The focus group will be recorded using an audio recorder so that we will not have to write down all your answers and we will not miss any of your useful comments. There are no 'right' or 'wrong' answer.

Explain you are starting the **audio recorder**.

Time interview started: ________________________

Are there questions about the training?

Clarify any questions that came up during the presentation.

Now, we would like to hear, what you think about the presented training.

→ Discussion about the training (see exemplary questions)

Time interview ended: ____________________________

Additional information

Presentation of the training manual:

If possible, the training manual should be sent to participants in advance. Also, at the beginning of the Focus Groups it should be presented by the researchers. We will conduct a Power Point presentation, which will include:

I. Presentation on the proposed training including:
   - Number of participants
   - Trainers and classroom support (coproduced with one professional and one peer trainer)
   - Learning style (presentations followed by exercises to practice skills and problem solve, time for discussion and questions)
   - Content of each day
   - Reading materials and exercises

Additionally, there will be a **hand out** and a **poster** with the key points of the presentation for the participants to take notes and as a reminder during the discussion.

Exemplary Questions to ask, during the discussion:

What do you think about the training for Peer Support?

Could the training be held in this context?

What changes need to be made?

Are any core elements missing?

What needs to be done, to adapt the training in this local context?

Is there anything missing in the materials?

…
