# Supplementary material for: Development of the UPSIDES global mental health training programme for peer support workers: Perspectives from stakeholders in low, middle and high-income countries
Source: PLoS One. 2024 Feb 26;19(2):e0298315. doi: 10.1371/journal.pone.0298315 (PMC10896522; doi:10.1371/journal.pone.0298315)
Supplement: S2 File — (PDF) [file pone.0298315.s002.pdf]

**Development of the UPSIDES global mental health training programme for peer support workers in low, middle and high-income countries**

Data analysis of focus group transcripts

Complete list of codes

| Code Nr. | Study Sites<br>Dar es Salaam (Tanzania) | Ulm I (Germany) | Ulm II (Germany) | Pune (India) | Beer Sheva (Israel) | Kampala (Uganda) | Hamburg (Germany) |
|---|---|---|---|---|---|---|---|
| 1 | cultural dynamics | accompanying | mentor | routine life | service provider | encouraging | define |
| 2 | confined | difficult situation | all-round companion | ideal PSV | service user | recover | group experience |
| 3 | recovery stage | counselling setting | overwhelmed | responsibilities | tell him | medication | recovery approach |
| 4 | class | freedom | knowledge from experience | recovery plan | mediate | gone through | common ground |
| 5 | engage | change of perspective | self-empowerment | rehabilitated in the society | recovery measure | experience | too short |
| 6 | stage | distance | terminology | social inclusion | protect | concentration | basic |
| 7 | stigma | access authorization | appendix | improvement | knowledge | time | transnational idea |
| 8 | community | recovery | stable | assess | understand | extension | low-threshold |
| 9 | stable | tell their own story | suitable for group work | medication | role models | community | demarcation |
| 10 | environment | breaks | ability to communicate | patient | familiarity | facilitation | target groups |
| 11 | outpatient | self-image | role-plays | own goal | ability to disclose | other work | professional recognition |
| 12 | inpatients | changing roles | target group | maintained | neutral, third party trainer | empowering | message |

**Development of the UPSIDES global mental health training programme for peer support workers in low, middle and high-income countries**

| | | | | | | | |
|---|---|---|---|---|---|---|---|
| 13 | goal of peer support sessions | sink in | draw it out | continuity | ups and downs | roles | clarity |
| 14 | method used for recovery | selection | trigger own developments and reflections | refresher | awareness | recovery | market |
| 15 | psychosis | motivated | regenerative elements | psychiatric examination | accept myself | support | quality assurance |
| 16 | pattern of the same | certain procedure | where peer support should be provided | behavioral interventions | share | commitment | Recovery College |
| 17 | follow up clinic | work through the manual | budget | mentally ill | imprisoned within the mental health system | engaged | preventive |
| 18 | rejection | gather experience | basic skills | mental health problem treatment | medication | excuses | already going on |
| 19 | group therapy | tight time period | service providers in advance | knowlegde | right | complete | translate |
| 20 | communication skills | chemistry | internship | sick | shared decisions | relapse | competitive |
| 21 | relapse | directive work | distinction | tension | time | interviews | maturing, co-developing, encounters, reflection |
| 22 | patients | communication | what they offer beyond | employment | self-defense | diligent | functionality |
| 23 | simple and clear | tandem | complement | setting goals | self-disclose | scares them away | competition with skilled workers |

| | | | | | | | |
|---|---|---|---|---|---|---|---|
| 24 | problem solving | flash light | competition | practical | pioneer | certificates | role model |
| 25 | ground rules | distance and self-protection | suggestions | woman | training for the places that are hiring peers | graduation | citizen help |
| 26 | time | dreams, goals and planning | developing realistic goals | marriage | peer professional-defining | market | churches |
| 27 | | transparency | reveal themselves, as peers | goals should be given | drawer policy | pushing | distress |
| 28 | | goal-oriented | supervision | role play | tool of assistance | motivation | large scale of offers |
| 29 | | new perspective on the illness | transparent | staff members | summary | message | groups |
| 30 | | attitude | professionals have fears | counseling sessions | reduce their use of medication-training | formally invited | profiling battles |
| 31 | | stand up for your rights | separate information event | stability | rehabilitation services, social security, labor rights | carers | recovery evaluation |
| 32 | | confrontation with one's own recovery history | confidentiality | working really well | simulations | permission | slimmed down, incredibly fast procedure |
| 33 | | selection criteria | cooperate | level | self-stigmas | allow | academic recovery |

**Development of the UPSIDES global mental health training programme for peer support workers in low, middle and high-income countries**

| | | | | | | |
|---|---|---|---|---|---|---|
| | | | | | | accompaniment and the non-academic |
| 34 | human being | data protection | hospital policies, procedure for complaint and admission procedure | they felt bad for me | financially and socially | self-empowerment |
| 35 | development opportunities | unbiased | PSVs are not staff | stigmas | disseminate | justice |
| 36 | inspiration | group identity | specific | "them" | family or our caretakers | community support |
| 37 | personality development program | discreetly | related to patients | coping | know | feel strange |
| 38 | follow-up programmes | own limits | criteria | take me seriously | triggers | coming out of the closed |
| 39 | unleash someone on people who can't fight back | convince | education | hostility | formal document | allocation |
| 40 | business | collegial counselling | communication skills | advocation | keep time | become expert knowledge from experience |
| 41 | medication | supervisors from other institutions | code of conduct | strategy | extended | recovery café |
| 42 | context that I can question things | network | community | connect | minimum level of education | self-help |

**Development of the UPSIDES global mental health training programme for peer support workers in low, middle and high-income countries**

| | | | | | | |
|---|---|---|---|---|---|---|
| 43 | scope | join forces | guardians | orientation for new employees | English | anti-stigmatization |
| 44 | supervision | been patients here in the hospital | parents | backbone | literate | door openers |
| 45 | resistance | stigmatize | involve the family | approach extreme circumstances | labeling and discriminating | who pays them, how does they pay |
| 46 | sandwich leader | travel costs | caregivers | mental illness | level of recovery | stigma |
| 47 | stable | creative peer work | motivation | chemical imbalance | panel of 2-3 people | isolation |
| 48 | employment law | loneliness | expectations | identify | model of care | dichotomy |
| 49 | assign a patient to someone | arranging everyday life | Are they involved in talking more or does work actually get done? | normal reaction | re-arrange | family helpers |
| 50 | different clinical pictures | guidelines | job description | open dialogue | active listening, communication | professional license |
| 51 | learning from each other | ressources-oriented, empowerment-oriented approach | allotted other work | shell-shock, post trauma, war | level the ground | refugee issue |
| 52 | development incentives | go out and do something | encourage | peers who are willing to | recovery language | trauma |

| | | | | | |
|---|---|---|---|---|---|
| | | | work in this job | | |
| 53 | fundamental way of working | frame | group support | refining your goals | coaching |
| 54 | individual component | problem | salary | tools | consulting |
| 55 | negotiation | supervising | flexible with needs | conflict resolution | pure peer character |
| 56 | combination | review of the work | | wellness plan | biographical work |
| 57 | non-violent conversation, different tools for language | close watch | | assure | doesn't have to be above the other |
| 58 | meta-model | training for staff | | valuable ressources | network |
| 59 | take care of themselves | | | limits | |
| 60 | group work | | | planning ahead | |
| 61 | role model | | | alternatives | |
| 62 | open offer | | | fall back position | |
| 63 | distinguishes | | | delivering | |
| 64 | | | | local examples | |
| 65 | | | | African time | |
| 66 | | | | breakfast | |
| 67 | | | | strictly | |
| 68 | | | | transport refund | |
| 69 | | | | simple rules | |

depending
screening
lazy
biased
force
sick role
time management
allowance
diagnosis
rest
refresh
range
energizing exercises
handouts
role plays
psychiatric language
