# Supplementary material for: Development of the UPSIDES global mental health training programme for peer support workers: Perspectives from stakeholders in low, middle and high-income countries
Source: PLoS One. 2024 Feb 26;19(2):e0298315. doi: 10.1371/journal.pone.0298315 (PMC10896522; doi:10.1371/journal.pone.0298315)
Supplement: S3 File — (PDF) [file pone.0298315.s003.pdf]

Relevant contents and methods for training

Topics for training

communication
communication skills
communication skills
non-violent conversation,
different tools for language
active listening, communication

hospital policies, procedure for
complaint and admission procedure
ability to disclose drawer policy
reveal themselves, as peers
tell their own story
biographical work
confrontation with one's
own recovery history
coming out of the closed
personality development program

conflict resolution
problem solving

reduce their use of
medication- training

self-empowerment self-image
self-empowerment

stand up for your rights
right
advocation
justice
trauma
own limits
code of conduct guidelines
take care of themselves
ground rules
distance
distance and self-
protection
goal of peer support
sessions
roles job description

dreams, goals and planning
setting goals
own goal goal-oriented
refining your goals
developing realistic goals
development opportunities

rehabilitation services,
social security, labor rights

open dialogue

group work model of care

recovery recovery
method used for recovery
recovery language
recovery plan
wellness plan
routine life
recovery approach

behavioral interventions
mental health problem treatment
meta-model

fundamental way of working
common ground
level the ground
message message

basic skills
knowlegde knowledge
tools

different clinical pictures

TANZANIA INDIA UGANDA ISRAEL Hamburg Ulm 1 Ulm 2

Methods for training

strictly simple rules specific
basic practical simple and clear
breaks regenerative elements

role-plays role plays role play
summary simulations
maturing, co-developing,
encounters, reflection

strategy
handouts
energizing exercises

trigger own developments
and reflections
learning from each
other
group experience group identity
flash light

Adjustments

appendix re-arrange
local examples
refresher refresh
maintained

Factors of local implementation

Access requirements

screening interviews assure
panel of 2-3 people
separate information event
formally invited
disseminate market
selection criteria
selection criteria
level
level of recovery
education
minimum level of education
literate
concentration
stability
stable
stable
peers who are willing to work in this job
ability to communicate
motivation
motivated
motivation
engaged
complete commitment
suitable for group work
expectations
pushing force
allowance
permission
allow

Challenges in implementation

Limited ressources

valuable ressources limits
alternatives
fall back position
transport refund
travel costs
planning ahead
facilitation
environment
English
breakfast rest
development incentives

Employment

employment
certificates
formal document
graduation
professional license
employment law
access authorization
budget
salary
business
who pays them, how does they pay
certain procedure
data protection
confidentiality

Temporal implementation

time time time
frame range
extended extension
continuity
draw it out
slimmed down, incredibly fast procedure
tight time period
keep time
time management
too short
overwhelmed
sink in
delivering

Involvement of the local environment

already going on
join forces
clarity market
large scale of offers
difficult situation
network
network
carers
caregivers
parents
family or our caretakers know
involve the family

Work support

training for the places that are hiring peers
training for staff
supervision
supervising
supervision
supervisors from other institutions
collegial counselling
gather experience
orientation for new employees
service providers in advance
internship
neutral, third party trainer
tandem
flexible with needs
tool of assistance

TANZANIA INDIA UGANDA ISRAEL Hamburg Ulm 1 Ulm 2

Attitude towards PSWs

Expectations for Peer Support Workers

ideal PSV

working really well

become expert knowledge from experience

transparency

pioneer

sandwich leader

Reservations towards peer support workers

Are they involved in talking more or does work actually get done?

unleash someone on people who can't fight back

been patients here in the hospital

excuses

lazy

diligent

sick

relapse

related to patients

assign a patient to someone

scares them away

Monitoring mechanisms

assess

review of the work

close watch

psychiatric examination

counseling sessions

tell him

goals should be given

quality assurance

Difficulties in working together as a team

take me seriously

they felt bad for me

resistance

combination

complement cooperate

staff members problem

biased

competition

"them"

self-defense

self-disclose

tension

hostility

profiling battles

competition with skilled workers

competitive

professional recognition

doesn't have to be above the other

PSVs are not staff

allotted other work

other work

professionals have fears

TANZANIA INDIA UGANDA ISRAEL Hamburg Ulm 1 Ulm 2

Task description for PSWs

Role definition

guardians
mentor
all-round companion

Tasks

consulting
mediate
anti-stigmatization
coaching
go out and do something
accompanying
encourage
connect
engage
support
understand
encouraging
shared decisions
counselling setting
share

assumed mechanisms of impact

inspiration
door openers
role models role model role model
gone through experience
familiarity
empowering
preventive
low-threshold
transparent
change of perspective
changing roles
attitude

responsibilities
work through the manual
backbone
protect

Attitude in Peer Support

individual component
creative peer work
freedom
open offer
chemistry allocation
context that I can question things
negotiation
directive work
discreetly
suggestions
convince
knowledge from experience
unbiased
ressources-oriented, empowerment-oriented approach

Demarcation from other mental health professionals

demarcation distinction
peer professional- defining
terminology
academic recovery accompaniment and the non-academic
distinguishes
pure peer character
what they offer beyond
translate
define

Target groups

group support groups
target groups
group therapy
target group
scope
where peer support should be provided
self-help
citizen help
community support
churches
Recovery College
recovery café
family helpers
refugee issue

TANZANIA INDIA UGANDA ISRAEL Hamburg Ulm 1 Ulm 2

Understanding of MH conditions

Recovery-oriented approach

identify human being
accept myself
approach extreme circumstances
normal reaction
ups and downs
awareness feel strange
distress

recover recovery measure
recovery stage recovery evaluation
improvement

transnational idea
pattern of the same

Recovery-oriented approach

diagnosis
psychosis
confined
medication medication medication medication
chemical imbalance
functionality
dichotomy

Prognosis

relapse
triggers
coping
stable

Mental health care system

inpatients outpatient
follow up clinic
psychiatric language
imprisoned within the mental health system

Labeling of people in MH conditions

service provider
service user
patients patient
mentally ill
mental illness
sick role
new perspective on the illness

TANZANIA INDIA UGANDA ISRAEL Hamburg Ulm 1 Ulm 2

Difficulties for people in mental health crises / living with MH conditions

Social inclusion

social inclusion

loneliness

isolation

arranging everyday life

Discrimination

stigma stigmas stigma

stigmatize

self-stigmas

rehabilitated in the society

rejection

labeling and discriminating

economic disadvantages

financially and socially

depending

TANZANIA INDIA UGANDA ISRAEL Hamburg Ulm 1 Ulm 2

Characteristics of society

Structure of society

cultural
dynamics

community
community
community

class

stage

marginalised groups

woman

marriage

Local specifics of the society

shell-shock, post trauma,
war

African time

TANZANIA INDIA UGANDA ISRAEL Hamburg Ulm 1 Ulm 2
